# Explore the potential mechanism of Huachansu injection against osteosarcoma via metabolomics, network pharmacology and bioinformatics

**DOI:** 10.1186/s13020-025-01179-x

**Published:** 2025-08-05

**Authors:** Jingjing Meng, Xiangqi Zhang, Danfeng Xiang, Hanlu Liang, Shuai Zhao, Lingyan Xu, Jiao Yang, JunJun Chen, Jingxian Zhang, Yonglong Han

**Affiliations:** 1 Department of Pharmacy, Shanghai Sixth People’s Hospital Affiliated to Shanghai Jiao Tong University School of Medicine, Shanghai, 200233 China; 2 College of Food Science and Technology, Shanghai Ocean University, Shanghai, 201306 China

**Keywords:** Osteosarcoma, Huachansu injection, Metabolomics, Network pharmacology, Lipid metabolism, BCYRN1-miR-27a-3p-HMGCR axis

## Abstract

**Aim:**

Huachansu injection (HCSI) shows effective medicinal functions against osteosarcoma. This study aimed to reveal the underlying mechanisms of HCSI against osteosarcoma by integrating metabolomics, network pharmacology and bioinformatics.

**Methods:**

Metabolomics was used to identify different metabolites and pathways. Network pharmacology was utilized to predict the potential targets of HCSI against osteosarcoma. Differentially expressed lncRNAs and miRNAs were screened and the corresponding lncRNAs-miRNAs-mRNAs network were constructed through the GEO database and miRcode database. Machine learning and immune infiltration analysis were performed on the key target obtained from the intersection of network pharmacology and bioinformatics. The binding affinity between active compounds of HCSI and potential targets was evaluated by molecular docking. The underlying mechanisms were further validated by RT-qPCR and immunoblotting.

**Results:**

Lipid metabolism pathways were obtained by non-target metabolomics enrichment. A total of 44 HCSI targets associated with osteosarcoma were collected by network pharmacology. Intersection of the mRNAs obtained from ceRNA network with the above 44 targets yielded eight common targets. The main target HMGCR were obtained by machine learning and RT-qPCR. The BCYRN1-miR-27a-3p-HMGCR axis was subsequently screened as the primary ceRNA regulatory network in HSCI against osteosarcoma. Molecular docking also showed an excellent affinity between the active compounds of HCSI and HMGCR. In vitro experiments demonstrated that HCSI down-regulated HMGCR, thereby reduced intracellular cholesterol levels, and ultimately promoting osteosarcoma cell apoptosis.

**Conclusion:**

HCSI could inhibit osteosarcoma progression by regulating lipid metabolism through BCYRN1-miR-27a-3p-HMGCR axis, indicating that HCSI may provide insights for developing herbal medicine injection-based therapies for osteosarcoma.

**Graphical Abstract:**

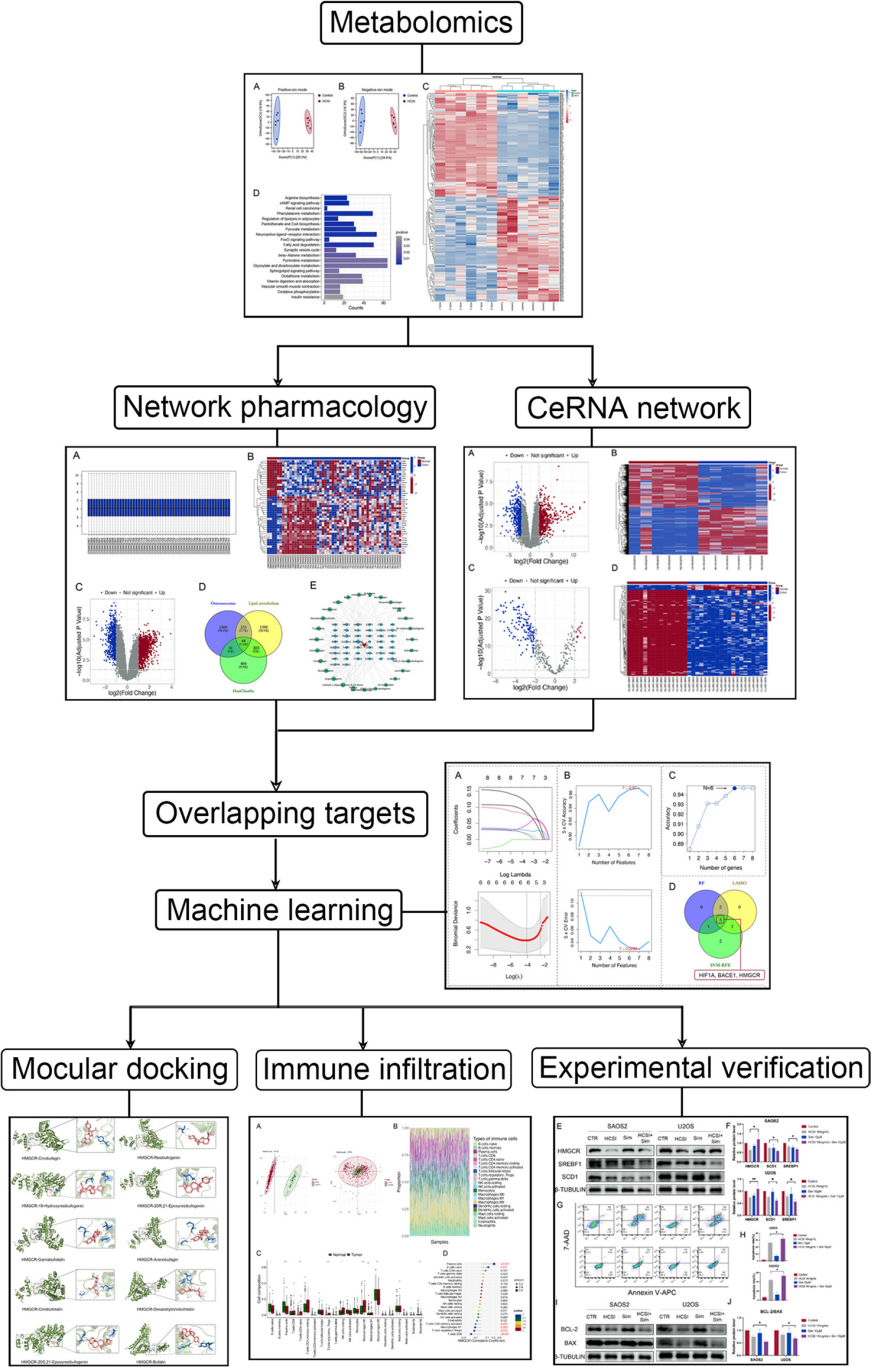

**Supplementary Information:**

The online version contains supplementary material available at 10.1186/s13020-025-01179-x.

## Introduction

Osteosarcoma is the most prevalent primary bone malignancy, accounting for approximately 35% of all malignant bone tumors, and occurs predominantly in adolescents and the elderly [1]. The current standard treatment for osteosarcoma is neoadjuvant chemotherapy (preoperative), surgical resection and adjuvant chemotherapy (postoperative) [2]. However, to date, the 5-year overall survival rate for patients with primary osteosarcoma is 60–75% and drops dramatically to 20–30% for patients with pulmonary metastases [3], which remain the leading cause of death in osteosarcoma [4]. Therefore, it is essential to develop adjuvant therapies that target the molecular basis of osteosarcoma cell proliferation, invasion and migration and improve patient survival [5].

Recent studies have shifted the focus to metabolic reprogramming in cancer cells, providing new avenues for therapeutic intervention in cancer [6]. Among the various metabolic pathways, lipid metabolic reprogramming is considered to be a new hallmark of malignancy and plays a key role in tumor development and therapy. Lipid metabolism in most tumor cells is usually aberrantly activated to promote rapid proliferation, invasion and resistance to apoptosis [7, 8]. In osteosarcoma, the role of dysregulated lipid metabolism has received attention. It has been found that lncRNA RPARP-AS1 could enhance the expression of key lipogenic enzymes and modulate the levels of free fatty acids and triglycerides by regulating the Akt/mTOR pathway, thereby promoting the progression of osteosarcoma cells [9].

Previous studies have confirmed that Chinese medicines, which showed the advantages of low toxicity, low side effects and high drug tolerance, are being developed as new cancer therapeutic drugs [10, 11]. Huachansu injection (HCSI) is refined from the sterile aqueous extract of the skin of the Bufo siccus [12]. It has been approved by the State Food and Drug Administration of China (ISO9002) for clinical oncology treatment [13]. The main active compounds of HCSI include cinobufotalin, cinobufagin, telocinobufagin, gamabufotalin, arenobufagin, bufotalin, resibufagenin and bufalin [14]. Numerous studies have shown that HCSI can affect the proliferation and metastasis of cancer cells and induce apoptosis by regulating cellular metabolism [15]. It has been reported that HCSI inhibits cholesterol metabolism by targeting the AMPK/SREBP1/FASN pathway and affects the tumor microenvironment, thus inhibiting the progression of hepatocellular carcinoma [16]. The anti-osteosarcoma effects of the primary bioactive components of HCSI, such as cinobufagin [17], bufalin [18], bufotalin [19], etc., have been reported. However, the anti-osteosarcoma effects of HCSI and its underlying mechanism have been less addressed.

In this study, we integrated metabolomics, network pharmacology and bioinformatics methods to explain the action mechanism of HCSI against osteosarcoma. Metabolomics can identify action targets and pathway networks by examining the dynamics of pathological metabolites in conjunction with network pharmacology [20]. In addition, combining network pharmacology with bioinformatics can explore disease intervention mechanisms from a broader perspective. Currently, the ceRNA hypothesis, which describes the interactions between RNA molecules, is a promising theory about the pharmacological mechanisms of traditional Chinese medicines (TCM) formulas. This hypothesis suggests that noncoding RNAs such as mRNA and lncRNA competitively bind the same miRNA through miRNA response elements (MREs), thereby communicating with each other and regulating their expression levels [21, 22]. Introducing ceRNA research into the field of cancer treatment in TCM will shed light on the deeper level of the pathogenesis of related cancers and the mechanism of action of TCM formulas [23]. In this study, we determined the direction of lipid metabolism by metabolomics. Based on the network pharmacology and ceRNA hypothesis, we constructed the lncRNA-miRNA-mRNA axis which may be modulated by HCSI to inhibit the progression of osteosarcoma through the regulation of lipid metabolism. Moreover, experimental validation was performed, which provided the basis for the subsequent anti-osteosarcoma study of the HCSI through lipid metabolism.

## Materials and methods

### Reagents

Huachansu injection comes from Anhui Huarun Jinzhan Pharmaceutical Co. Ltd (Huaibei, China 210,101–1) and is available in 5 ml (500 mg/ml) each. Dulbecco's Modified Eagle's medium (DMEM) was provided by Gibco (New York, USA). Fetal bovine serum (FBS) was provided by Bioscience (Shanghai, China). The penicillin/streptomycin solution and ProtLytic phosphatase inhibitor mixture were acquired from NCM Biotech (Suzhou, China). Phosphate buffered saline was obtained from Servicebio (Wuhan, China). Cell Counting Kit-8 (CCK-8) and BODIPY 493/503 were purchased from Beyotime (Shanghai, China). Total Cholesterol and Cholesteryl Ester Fluorometric Assay Kit was ordered from Elabscience (Wuhan, China). RIPA lysis buffer was obtained from Solarbio (Beijing, China). Sinvastatin was purchased from MedChemExpress (New Jersey, USA). APC Annexin V/7-AAD Apoptosis Detection Kit was purchased from Biolegend (San Diego, USA). EZ-press RNA Purification Kit and the Color Reverse Transcription Kit with gDNA Remover were obtained from EZBioscience (Roseville, USA). A protein-free blocking buffer and a chemiluminescence detection kit were purchased from Epizyme (Shanghai, China).

### Metabolomics

Prepared 12 dishes of SAOS2 cells with approximately 1 × 10^7^ cells per dish. After drug treatment, 1 mL of PBS was added, then the scraped cells were collected into a centrifuge tube and centrifuged at 1000 rpm for 1 min. Then the supernatant was removed and the precipitate was frozen in liquid nitrogen for 15 min. ﻿Add 1 mL of methanol: acetonitrile: water (2:2:1, v/v/v) to each sample, and vortex for 30 s. ﻿Immerse the sample in liquid nitrogen for rapid freezing for 5 min and take out the centrifuge tube. Then put it into a high-throughput tissue grinder (Jingxin, TL-48R) at 55 Hz for 60 s. Centrifuge at 12,000 rpm for 15 min and concentrate to dryness under vacuum. Add 150 μL of 50% methanol (containing 5 ppm 2-chlorophenylalanine) and vortex for 30 s. Centrifuge at 12000 rpm and 4 °C for 10 min, filter the supernatant through a 0.22 μm filter membrane, and add the filtrate to the test bottle. Take 10–20 μL of each sample filtrate and mix them into a QC sample for evaluating instrument stability and data reliability. ﻿An ACQUITY UPLC HSS T3 column (100 Å, 1.8 µm, 2.1 mm × 100 mm) of ﻿Ultra-high performance liquid chromatography (﻿Thermo, ﻿Vanquish Flex) was used with a flow rate of 0.4 mL/min, a column temperature of 40 ℃, an autosampler temperature of 8℃, and an injection volume of 2 μL. ﻿Thermo Orbitrap Exploris 120 mass spectrometer was used to collect DDA mass spectrometric data in positive and negative ion modes. HESI source, spray voltage 3.5 kV/–3.0 kV, sheath gas 40 arb, auxiliary gas 15 arb, capillary temperature 325 ℃, auxiliary, gas temperature 300 ℃, primary resolution 60,000, scan range 100–1000 m/z, AGC Target Standard, Max IT 100 ms, the top 4 ions were screened for secondary fragmentation, dynamic exclusion time was 8 s, HCD collision energy 30%, AGC Target Standard, Max IT Auto. All formal samples and QC sample were loaded into the equipment according to the above-mentioned chromatography and mass spectrometry methods.

### Active components and prediction of the action targets of drugs

The main components of HCSI were derived from the skin of Bufo siccus. Searched for “chanpi” and “chansu” in the Herb database for the chemical compounds of HCSI (http://herb.ac.cn/). This database collected information from SymMap, TCMID, TCMSP, and TCM-ID databases. The SMILES structure of active compounds was obtained from the PubChem database (https://pubchem.ncbi.nlm.nih.gov/). The Lipsinki principles was used to further screen active compounds (molecular weight ≤ 500, lipid-water partition coefficient ≤ 5, number of hydrogen bond donors ≤ 5, number of hydrogen bond acceptors ≤ 10). To predict the potential targets of HCSI, the BATMAN database (http://bionet.ncpsb.org.cn/batman-tcm/index.php), SEA database (https://sea.bkslab.org) and the SwissTargetPrediction database (https://www.swisstargetprediction.ch/) were used.

### Differential gene analysis of the GEO dataset

We obtained transcriptome sequencing series GSE16088, GSE16091, GSE225588, and GSE65071 from the GEO (www.ncbi.nlm.nih.gov/geo/) database, which includes gene expression profiles of osteosarcoma and normal bone samples. Datasets of lncRNAs, miRNAs and mRNAs between the normal group and osteosarcoma group were analyzed by “limma” package of R language. Genes with adj. p value < 0.05 and |log2 (fold change) |> 2 were considered significantly differential expression. The volcano map and heatmap were created using the “pheatmap” and “EnhancedVolcano” R packages.

### GO and KEGG analysis

The following R packages (“ggplot2”, “GOplot”, “enrichplot” and “prg.Hs.eg.db”) were applied to analyze GO and KEGG pathway enrichment, and adj. p value < 0.05 was considered statistically significant.

### HCSI, lipid metabolism and osteosarcoma target network construction and ceRNA network construction

Visualized the target genes of HCSI’s active compounds, lipid metabolism and osteosarcoma using Venny 2.1.0 (https://bioinfogp.cnb.csic.es/tools/venny/), and considered the intersected target genes as candidate target genes. Input the overlapping targets and related components into Cytoscape 3.9.0 (https://cytoscape.org/). In the same way, Visualized the above target genes and the genes obtained from ceRNA network using Venny 2.1.0, and then submitted the overlapping targets and related miRNAs and lncRNAs into Cytoscape 3.9.0.

### Machine learning algorithms

To further screen candidate genes, three machine learning algorithms were applied. Least absolute shrinkage and selection operator (Lasso) regression is often used to filter variables in order to prevent overfitting [24]. It has been reported that Random Forest (RF) is a useful method for handling high-dimensional data, building predictive models and predicting the importance of each variable. In addition, the Support Vector Machine—Recursive Feature Elimination (SVM-RFE) algorithm is appropriate for small sample datasets due to its ability to remove redundant factors and retain only variables related to the outcome [25]. Genes identified from the intersection of the three algorithms were considered candidate biomarkers.

### Correlation analysis of the target gene expression with immune infiltration

CIBERSORT is a deconvolution algorithm widely used to label genomes of different types of immune cells in the microenvironment [26]. This study executed “CIBERSORT” package to assess the proportion of the immune cell from the osteosarcoma gene expression profile. The frequency and proportion of immune infiltration was plotted for each sample using the "ggplot2" package. Differences in the proportions of the 22 immune cell types between normal and osteosarcoma samples were compared using the Wilcoxon test, with p < 0.05 considered statistically significant, and presented by using a stacked histogram based on the "ggplot2" package. Finally, Spearman's rank correlation coefficient was performed for correlation analysis between target gene expression and infiltrating immune cell content, and p < 0.05 was considered statistically significant.

### Molecular docking verification

Molecular docking was used to verify the binding of active components and the key target. Downloaded the 2D structure of active compounds of HCSI from the PubChem database, imported into ChemBio3D 17.0 software for energy minimization optimization, and save in mol2 format. The three-dimensional crystal structure of the HMGCR was downloaded from the PDB protein database (https://www.rcsb.org/). Used PyMOL 3.0.3 software to remove water molecules and residual ligands, and then imported into Autodock Tools 1.5.7 software, saving the processed file in pdbqt format. Molecular docking between the active compounds and HMGCR was performed using Autodock Tools software, the docking pocket was determined and the binding energy was calculated. Visualized the docking results using PyMOL 3.0.3 software.

### Cell culture and treatment

The osteosarcoma cell lines SAOS2, 143B, U2OS and MG63 were acquired from the Cell Bank of the Committee on Preservation of Typical Cultures, Chinese Academy of Sciences. The cells were grown in a high-glucose DMEM medium supplemented with 10% fetal bovine serum and 1% penicillin/streptomycin solution. The cells were cultured at 37 °C with a 5% concentration of CO_2_ in a specialized cell incubator. For the subsequent experiments, cells were treated with HCSI (4, 8, 16 mg/mL) or 10 μM simvastatin dissolved in dimethyl sulfoxide.

### Cell counting Kit-8 (CCK-8) assay

Cell viability and cell proliferation were assessed by CCK-8 assay. SAOS2 and U2OS cells (5000 cells/well) were inoculated in 96-well plates and cultured for 24 h. After that, started drug treatment at concentrations of 0, 2, 4, 8, 16, and 32 mg/mL. After 24 h, discarded the liquid above, introduced 100 μL of DMEM solution with 10% CCK-8 into every well and incubated for an hour at 37 ℃. Then cell viability was determined by measuring the absorbance at 450 nm using an EPOCH multi-function microplate reader (BioTek, USA).

### Flow cytometry

Cell apoptosis was analyzed by flow cytometry using the APC Annexin V/7-AAD Apoptosis Detection Kit. Cells were seeded in a 6-well plate. The experimental groups were treated with 0, 4, 8 and 16 mg/mL HCSI for 24 h, while the control group was treated with complete culture medium. The suspended and adherent cells were collected. Mixed the cell suspension with 5 µL of APC Annexin V, 5 µL of 7-AAD viability dye and 100 μL of Annexin V binding buffer and incubated the cells at 25 ℃ for 15 min. Added another 400 µL Annexin V Binding Buffer. Used a CytoFLEX flow cytometer (BECKMAN COULTER, USA) for cell analysis to determine the proportion of cell apoptosis.

### Quantitative real-time polymerase chain reaction (qRT-PCR) analysis

Total cellular RNA was extracted by EZ-press RNA Purification Kit. Afterwards, the Color Reverse Transcription Kit alongside the gDNA Remover was used for converting RNA into cDNA. The qRT-PCR assay was carried out using 2 × Color SYBR Green qPCR Master Mix (ROX1 plus), primers, and ddH_2_O. Four replicates were set for each sample. The cycling parameters are as follows: 95 ℃ for 5 min, 95 ℃ for 10 s, 60 ℃ for 30 s, and 72 ℃ for 30 s. The transcript levels of mRNA and lncRNA were normalized to β-actin and GAPDH. The transcript levels of miRNA were normalized to U6. Supplementary Table 1 contains the listed primer sequences.

### Wound healing assay

SAOS2 and U2OS cells were cultured in 6-well plates until confluent, and wounds were created on the cell monolayer. The wound areas in real time and after 24 h of HCSI treatment were recorded by the optical microscope.

### Quantification of cellular neutral lipids

For observation of intracellular lipid accumulation, cells in 6-well plates were incubated with BODIPY 493/503 (10 μM) for 30 min at 37 °C. The fluorescent-labeled lipid droplets were then observed using a fluorescence microscope.

### Quantification of cholesterol

SAOS2 and U2OS cells with different treatment were lysed in RIPA buffer for 5 min. Centrifuged at 12,000 rpm for 5 min at 4 ℃. The above supernatant was used for the assay. The levels of cholesterol were determined with cholesterol kits according to the manufacturers’ protocols [27].

### Western blot

SAOS and U2OS were lysed in RIPA buffer. Protein samples were denatured in 5 × loading buffer, and then subjected to 10% SDS-PAGE gels. Transferred the gels onto the PVDF membranes (0.45 μm, Millipore Corporation, USA). The membranes were blocked for 30 min and probed with the appropriate primary antibodies overnight at 4 ℃. Then, the membranes were probed with anti-mouse or anti-rabbit immunoglobulin for 2 h. The signaling was visualized with the ECL detection kit in the ImageQuant LAS 4000 mini visualizer (GE Healthcare Bio-Sciences AB). The primary antibodies utilized are as follows: anti-Bax (A19684, ABclonal, China), anti-Bcl-2 (A19693, ABclonal, China), anti-SREBF1 (66875, Proteintech, China), anti-HMGCR (ET1702, Huabio, China), anti-SCD1 (2794 T, Cell Signaling Technology, USA), anti-Beta-Tubulin (66240, Proteintech, China).

### Statistical analysis

Statistical analysis was performed using GraphPad Prism 9.0 (USA). The experimental data were expressed as the mean standard deviation of three or more independent experiments. Differences between two groups were evaluated by T-test. One-way Analysis of Variance (ANOVA) was conducted to compare multiple groups with one independent variable followed by Bonferroni correction. A P-value < 0.05 was considered statistically significant.

## Results

### HCSI may affect osteosarcoma cell progression by regulating lipid metabolism

**Fig. 1:**
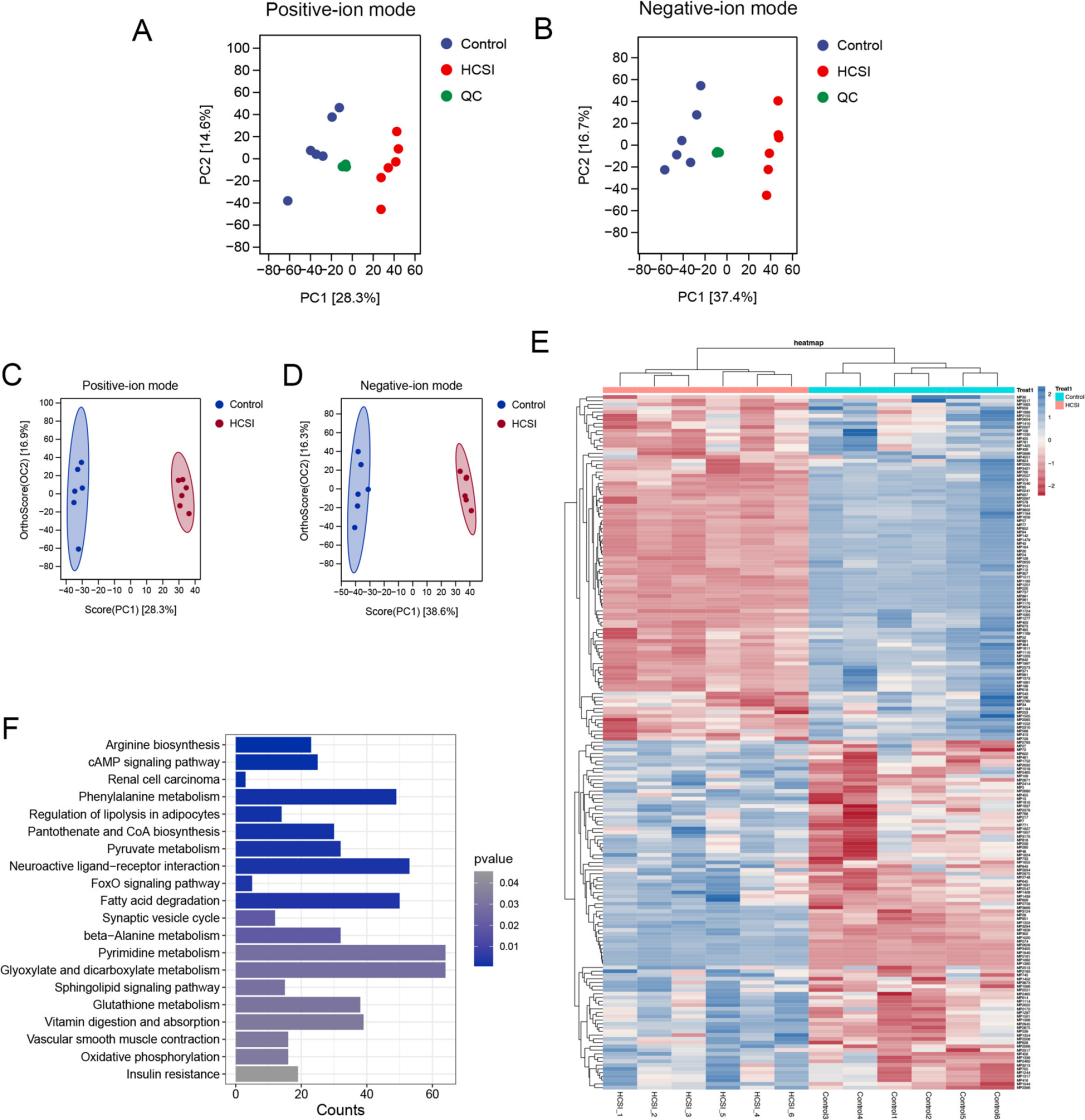
HCSI may affect osteosarcoma cell progression by regulating lipid metabolism. **A** PCA model of control group, HCSI group and QC group in positive- ion mode. **B** PCA model of control group, HCSI group and QC group in negative-ion mode. **C** OPLS-DA scores scatter illustrating differences between the control groups and HCSI treated groups in positive-ion mode. **D** OPLS-DA scores scatter illustrating differences between the control groups and HCSI treated groups in negative-ion mode. **E** Heat map of hierarchical cluster analysis of differential metabolites in positive-ion mode and negative-ion mode. **F** Enriched metabolic pathways of differential metabolites in cell samples

To further explore the mechanisms underlying HCSI efficacy in osteosarcoma treatment, non-target metabolomics were applied to examine metabolites in SAOS2 cells. The metabolomics data of all samples were modeled to observe the overall distribution and degree of aggregation of samples in the control, HCSI group and quality control (QC) groups. The QC samples in this study were densely distributed on the principal component analysis (PCA) plot both in positive-ion mode (Fig. 1A) and negative-ion mode (Fig. 1B), indicating good data quality. With the aim of differentiating groups and visualizing metabolic differences, orthogonal partial least squares discriminant analysis (OPLS-DA) was performed, using the global characteristics of the raw data (Fig. 1 C-D). The parameters R2Y and Q2 in OPLS-DA of the cell samples were 0.996, 0.929 and 0.995, 0.963 in positive and negative-ion mode, respectively. These results suggested that HCSI treatment resulted in significant metabolic changes. Through a combination of univariate and multivariate statistical analyses, a total of 289 differentially expressed metabolites with VIP > 1 and p < 0.1 in positive-ion mode and negative-ion mode were identified (Fig. 1E and Supplementary Table 3). In parallel, a KEGG enrichment analysis on all differential metabolites was performed. As shown in Fig. 1F, three pathways related to lipid metabolism were significantly affected, namely fatty acid degradation, regulation of lipolysis in adipocytes and sphingolipid signaling pathway. Key metabolites involved in these pathways included sphinganine, glutaric acid, palmitic acid and L-palmitoylcarnitine. The results suggested that lipid metabolism might be the primary pathway mediating the efficacy of HCSI in the treatment of osteosarcoma.

### Target identification of anti-osteosarcoma targets of Huachansu (HCS) through lipid metabolism

**Fig. 2:**
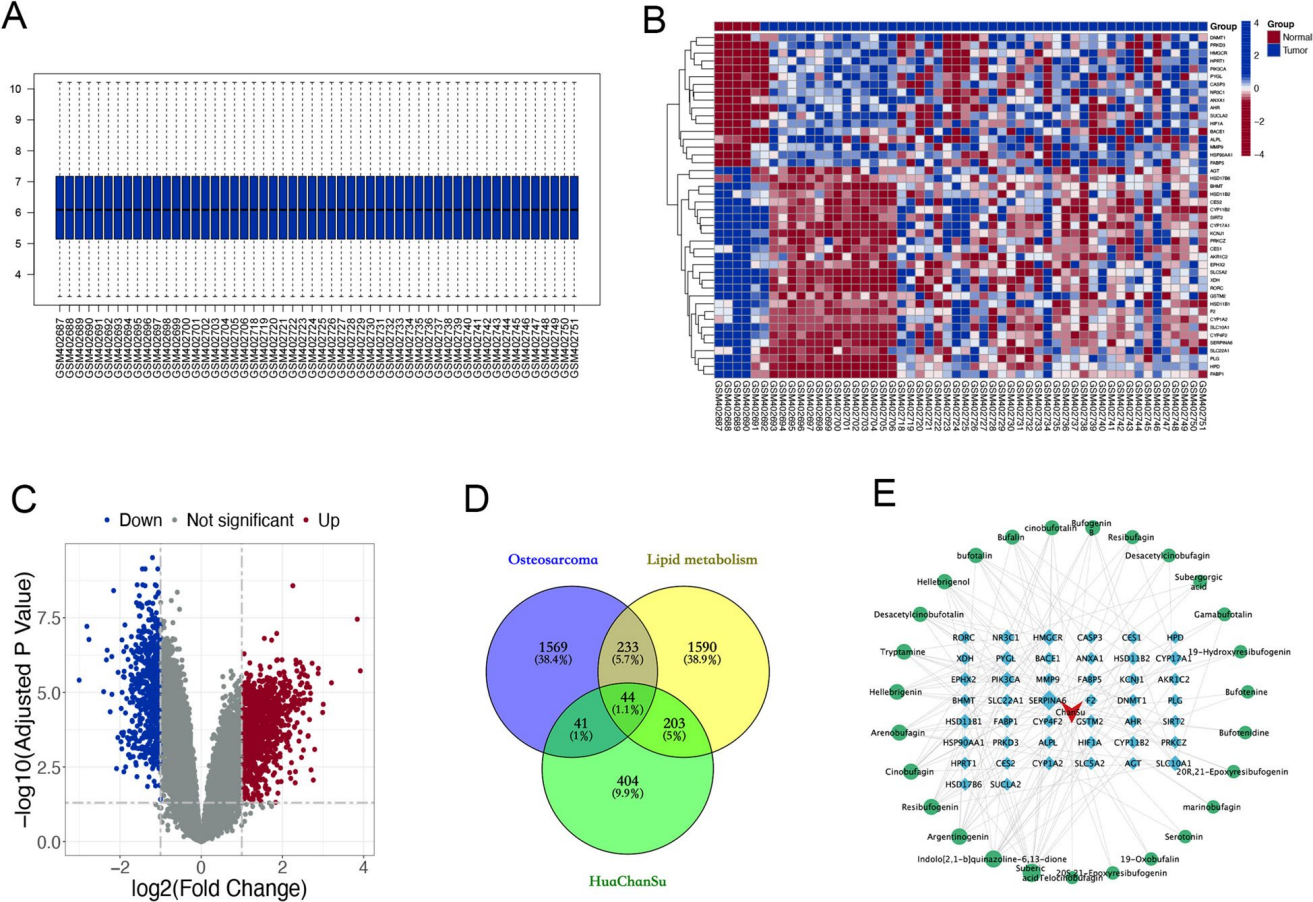
Target identification of anti-osteosarcoma targets of HCS through lipid metabolism. **A** Normalization box plot of the GSE16088 and GSE16091 datasets. **B** Heatmap displaying the 44 DEGs as well as the intersecting targets. **C** Volcano plot of the DEGs. Red represents upregulated genes, while green represents downregulated genes. **D** A Venn diagram of the intersection genes of HCS, lipid metabolism and osteosarcoma. **E** Construction of “drug-active compounds-targets” network diagram. Circle nodes active compounds, diamond nodes represent targets, and V nodes represent the drug

27 main compounds of HCS were obtained from HERB database, PubChem and swissADME after screening. 692 targets of the active compounds were searched via the BATMAN, SEA and SwissTargetPrediction databases. Based on GSE16088 and GSE16091 series, there were 1887 differentially expressed genes (DEGs) that fit |log2 (FC)|> 1 and p. adj < 0.05, of which 1192 were upregulated and 695 were downregulated (Fig. 2B, C). Through the GeneCards and GSEA databases, 1867 genes related to lipid metabolism were obtained after removing duplicates. Using Venny 2.1.0, a total of 44 overlapping genes were selected after intersection (Fig. 2D), indicating that HCS may regulate multiple targets to inhibit osteosarcoma by interfering with lipid metabolism. Afterwards, the network of 44 target genes was mapped against 27 core components of HCS using Cytoscape 3.10.1 (Fig. 2E).

### Differential expression of lncRNAs and miRNAs of osteosarcoma

**Fig. 3:**
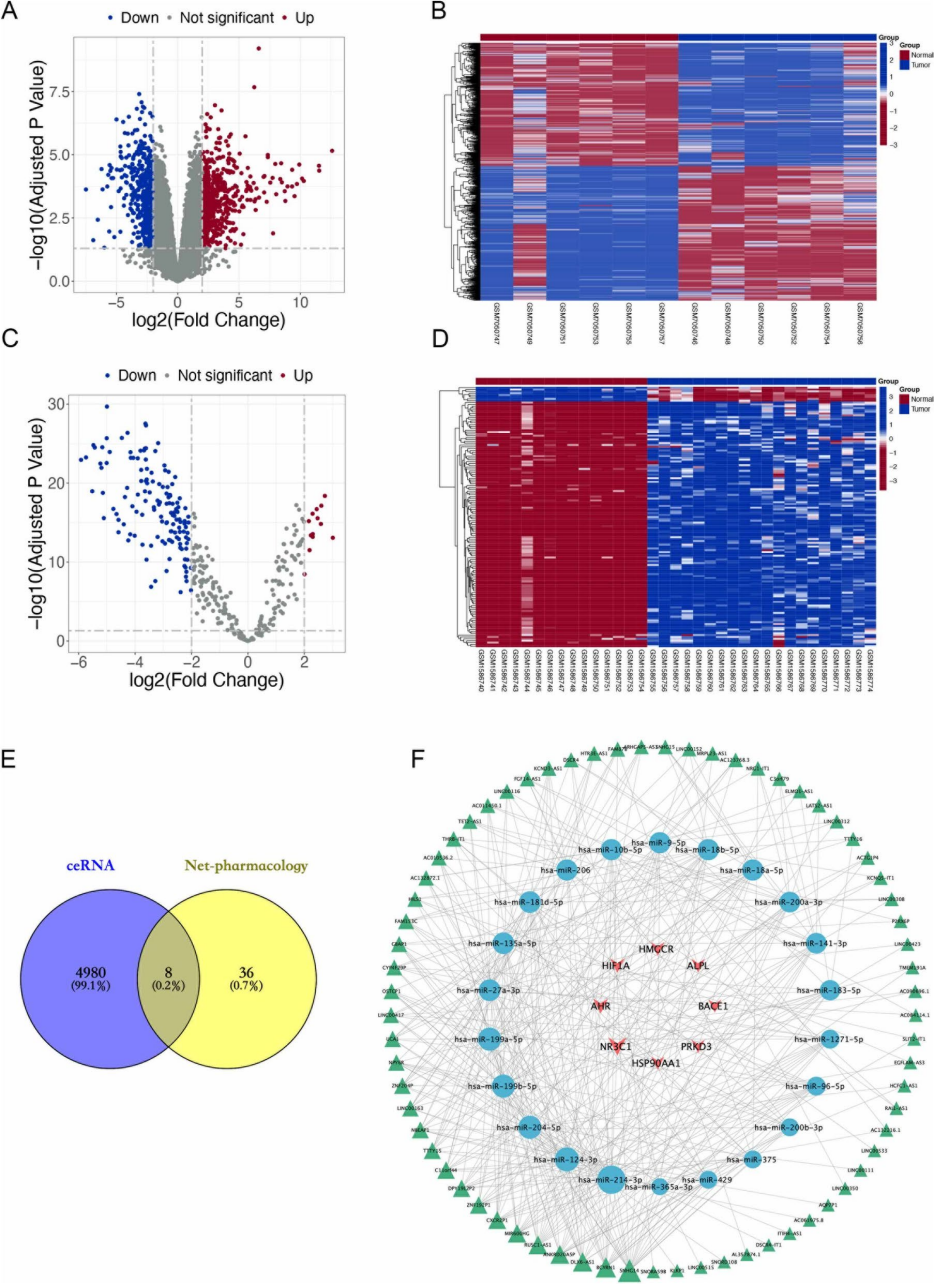
Differential expression of lncRNAs and miRNAs of osteosarcoma. **A** Volcano plot of the DElncRNAs in the GSE225588 series. **B** Heatmap showing the 993 DElncRNAs. **C** Volcano plot of the DEmiRNAs in the GSE65071 series. **D** Heatmap showing the 149 DEmiRNAs. **E** A Venn diagram of the intersection mRNA from ceRNA and network pharmacology. **F** Construction of “lncRNAs-miRNAs-mRNAs” ceRNA network diagram. Triangular nodes represent lncRNAs, circle represent nodes miRNAs, and V nodes represent mRNAs. Size represents the RNA's degrees, with larger sizes indicating higher degrees

After analyzing the GSE225588 series, 993 differentially expressed lncRNAs that met |log2 (FC)|> 2 and p. adj < 0.05 were obtained. Among them, 519 lncRNAs were significantly upregulated and 474 lncRNAs were significantly downregulated (Fig. 3A). According to the GSE225588 series, the expression of 993 lncRNAs in each sample was searched, with significant differential expression heatmaps was drawn (Fig. 3B). The miRcode database was then used. 197 miRNAs were predicted to be able to connect to differential lncRNAs. In the same way, by analyzing the GSE65071 series, 149 differentially expressed miRNAs that matched |log2 (FC)|> 2 and p. adj < 0.05 were found, of which 13 were upregulated and 136 were downregulated (Fig. 3C, D). Intersecting this with the results obtained from prediction gave 44 miRNAs.

### mRNA finishing and construction of ceRNA network

The 44 target miRNAs were retrieved from the starBase, miRanda and TargetScan databases, and a total of 4988 mRNA targets were obtained after intersection in the three databases. This was then intersected with the 44 genes obtained from network pharmacology. Eight genes were identified, including HMGCR, HIF1A, AHR, NR3C1, HSP90AA1, PRKD3, BACE1, ALPL (Fig. 3E). Subsequently, based on the results of integrating network pharmacology and bioinformatics, Cytoscape 3.10.1 was used to construct a ceRNA network containing 8 mRNAs, 22 miRNAs and 69 lncRNAs (Fig. 3F).

### Identification of the core target for HCS through machine learning

**Fig. 4:**
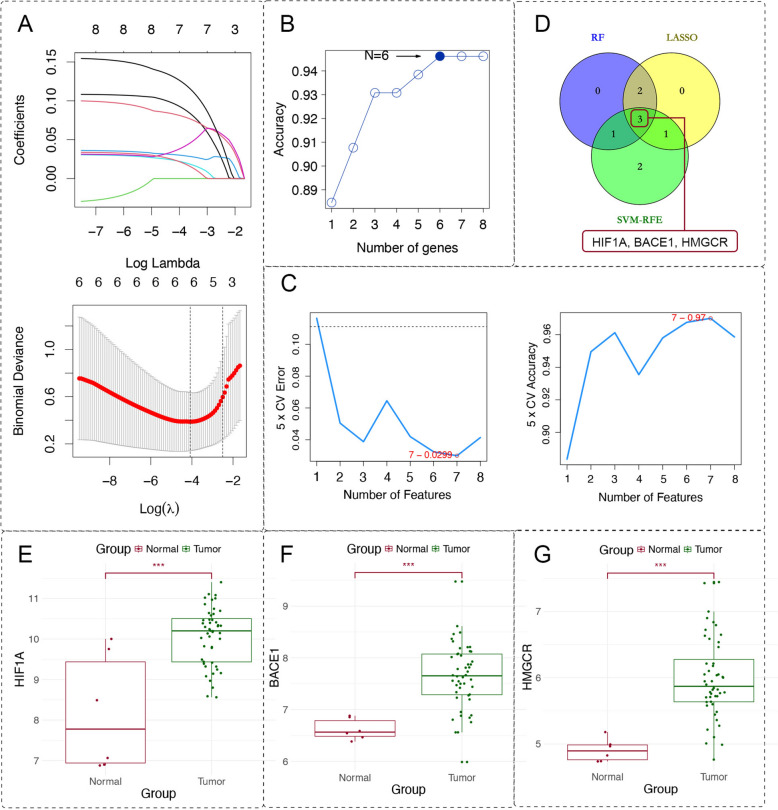
Identification of the core target for HCS through machine learning. **A** The coefficients and regularization diagrams from the LASSO logistic regression algorithm with tenfold cross-validation. **B** The accuracy rate curves based on the RF algorithm to select 6 related features. **C** The accuracy and error rate curves of fivefold cross-validation based on the SVM-RFE algorithm. **D** A Venn diagram of the intersection targets from 3 machine learning algorithms. **E**–**G** The box plots of the expression analysis of the three targets based on the GSE16088 and GSE16091 datasets

In order to further identify the core targets of the anti-osteosarcoma activity of the HCS, three machine learning algorithms (LASSO, SVM-RFE, and RF) were applied. Both the lasso algorithm (Fig. 4A) and the RF algorithm (Fig. 4B) filtered 6 genes from 8 targets. While the SVM algorithm screened 7 targets from them (maximum accuracy = 0.97, minimum RMSE = 0.0299) (Fig. 4C). Then, 3 overlapping genes, which includes HMGCR, BACE1 and HIF1A, were selected after intersection by using Venny 2.1.0 (Fig. 4D). After retrieving these three genes in the GSE16088 and GSE16091 series, it can be found that they are all significantly overexpressed in the cancer tissues of 48 osteosarcoma patients (Fig. 4E–G).

### Identification of the LncRNA-miRNA-mRNA axis

**Fig. 5:**
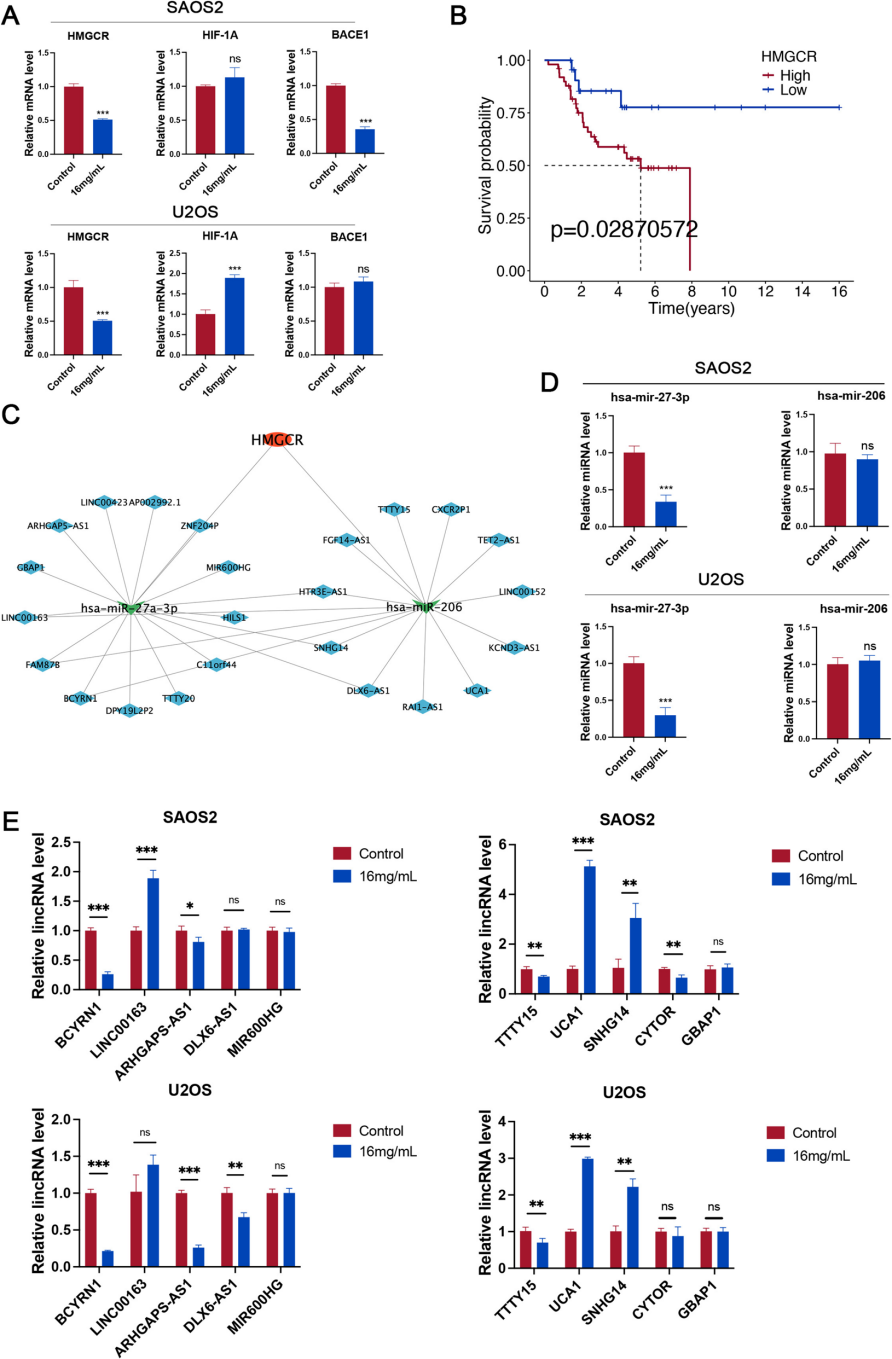
Construction of the LncRNA-miRNA-mRNA axis. **A** Quantitative RT-PCR analysis for expression of HMGCR, HIF-1A and BACE1 in SAOS2 and U2OS cells with HCSI treatment (n = 3). **B** Kaplan–Meier overall survival curve based on HMGCR mRNA expression in TARGET OS dataset. **C** Construction of the lncRNAs-miRNAs-HMGCR network. **D** Quantitative RT-PCR analysis for expression of miR-27-3p and miR-206 in SAOS2 and U2OS cells with HCSI treatment (n = 3). **E** Quantitative RT-PCR analysis for expression of 10 lncRNAs in SAOS2 and U2OS cells with HCSI treatment (n = 3). Data are presented as mean ± SD (n = 4). *P < 0.05, **P < 0.01, and ***P < 0.001 versus the Control group

To determine the LncRNA-miRNA-mRNA axis through which HCSI exerts its anti-osteosarcoma effects by regulating lipid metabolism, the expression of mRNAs (Fig. 5A), miRNAs, and lncRNAs obtained by enrichment was compared between the control group and the dosing group using RT-qPCR. HMGCR is significantly upregulated in 48 samples of osteosarcoma. Among the three target genes obtained by machine learning, only the expression of HMGCR is significantly downregulated in both cells after HCSI administration. It has been shown that HMGCR catalyzes the rate-limiting step in the synthesis of cholesterol, which plays an important role in the regulation of lipid metabolism *in vivo* [28]. We performed KM survival analysis on the pivotal target HMGCR. The results showed that patients with high expression of HMGCR had shorter overall survival (OS) compared to patients with low expression (Fig. 5B). High expression of HMGCR is considered to be a risk factor affecting the prognosis of osteosarcoma patients. Therefore, HMGCR was selected to establish the lncRNAs-miRNAs-HMGCR network for further analysis (Fig. 5C). This network includes 1 mRNA, 2 miRNAs and 24 lncRNAs. miR-27a-3p is significantly upregulated in patients with osteosarcoma by the GSE65071 series, whereas the expression of miR-27a-3p expression was reversed after HCSI administration (Fig. 5D). It has been reported that miR-27a-3p is involved in the process of lipid metabolism and can significantly regulate the expression of HMGCR and FASN, which is a key fatty acid synthase for fatty acid synthesis [29–31]. Several studies have shown that BCYRN1 expression is significantly increased in a variety of tumors and has been shown to be a biomarker for these tumors, including colorectal cancer [32], hepatocellular carcinoma [33], bladder cancer [34] and non-small-cell lung cancer [35]. The expression of BCYRN1 in osteosarcoma cells was significantly decreased after the treatment of HCSI (Fig. 5E). Therefore, we established BCYRN1-miR-27a-3p-HMGCR axis.

### Immune cell infiltration analysis

**Fig. 6:**
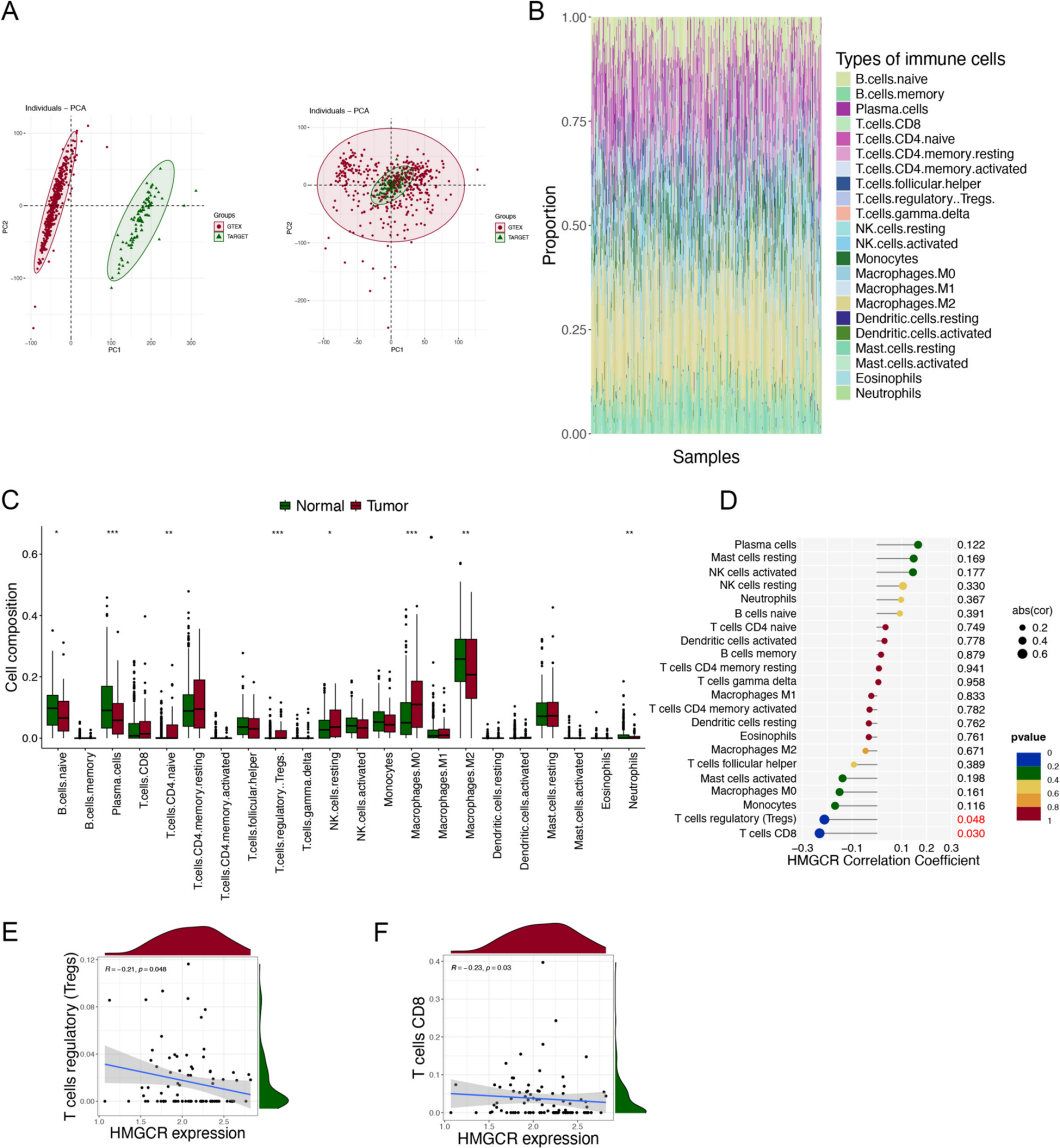
Immune cell infiltration analysis. **A** Eliminating the batch effect between GTEX and TCGA databases. **B** Stacked column chart of the infiltration of 22 types of immune cells in a total of 484 samples. **C** Box plot of the infiltration levels of 22 immune cells between the normal and osteosarcoma groups. **D** Lollipop graph of correlation between HMGCR expression and 22 immune cells in tumor tissues. **E**–**F** Correlations between HMGCR expression and immune cells (P < 0.05)

To further analyze the clinical significance of osteosarcoma-infiltrated immune cells, we used 396 normal muscle samples from the GTEX database and 88 osteosarcoma samples from the TCGA database. Figure 6B demonstrates the infiltration of 22 immune cell types within total 484 samples by using CIBERSORT. We then analyzed the distinctions in the infiltration of the different immune cells in the control and osteosarcoma samples and found that naive B cells, plasma cells and M2 macrophages were significantly highly expressed in the osteosarcoma samples, whereas CD4 naive T cells, T cell regulatory Tregs, resting NK cells and M0 macrophages were significantly less expressed in the osteosarcoma samples (Fig. 6C). In addition, we analyzed the potential relationship between the infiltration of immune cells in tumor issues and the expression level of HMGCR (Fig. 6D). The outcomes revealed that T cell regulatory Tregs and T cells CD8 were negatively associated with HMGCR (P < 0.05) (Fig. 6E, F). The above results suggest that HMGCR may influence the growth and metastasis of osteosarcoma by regulating the levels of tumor-infiltrating immune cells.

### Verification of molecular docking

**Fig. 7:**
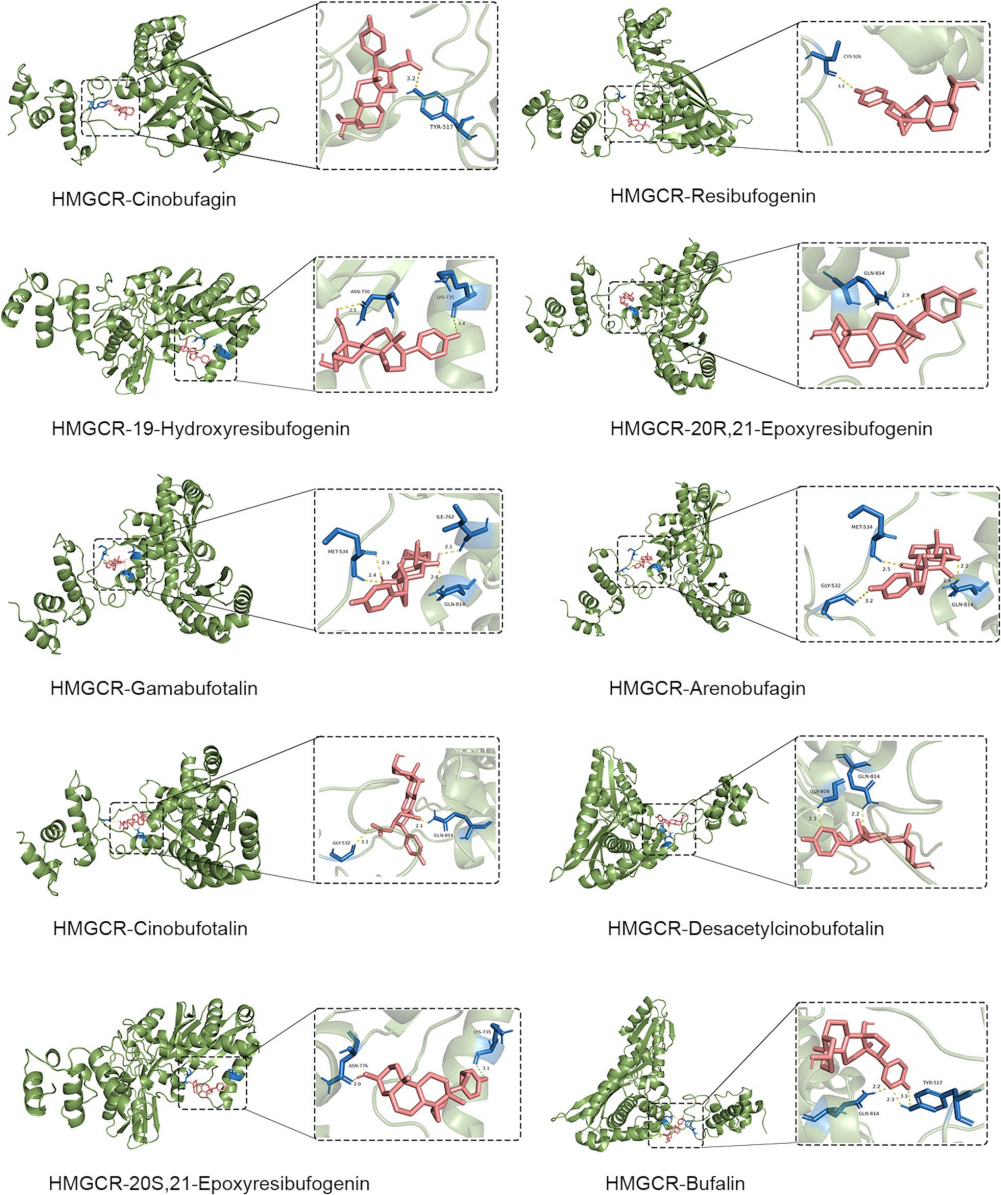
Verification of molecular docking. Visualization of molecular docking results of 10 compounds with HMGCR. The green portions represent the target protein HMGCR. The red portions represent the compounds. Hydrogen bond interactions between the ligands and the receptors are represented by the yellow dashed lines. And the blue portions represent the amino acid residues linked to hydrogen bonds

To validate the binding abilities of the active ingredients in HCS to the key target in osteosarcoma, we performed molecular docking with HMGCR using 27 monomers as ligands obtained from the network pharmacology. The binding energy of the 27 monomers to HMGCR is illustrated in the Supplementary Table 2. A lower binding energy means a better binding efficiency and a more stable interaction between the ligand and the core target protein. It can be observed that the binding energies of 20 ligand-receptor pairs are lower than − 7 kJ•mol^−1^, suggesting that HMGCR may be the prospective binding target of HCS in the treatment of osteosarcoma. PyMOL 3.0.3 was employed to visualize the interactions and binding modes of compounds and targets with the top 10 high free binding energy score (Fig. 7).

### HCSI inhibits the proliferation and migration of osteosarcoma cells in vitro

**Fig. 8:**
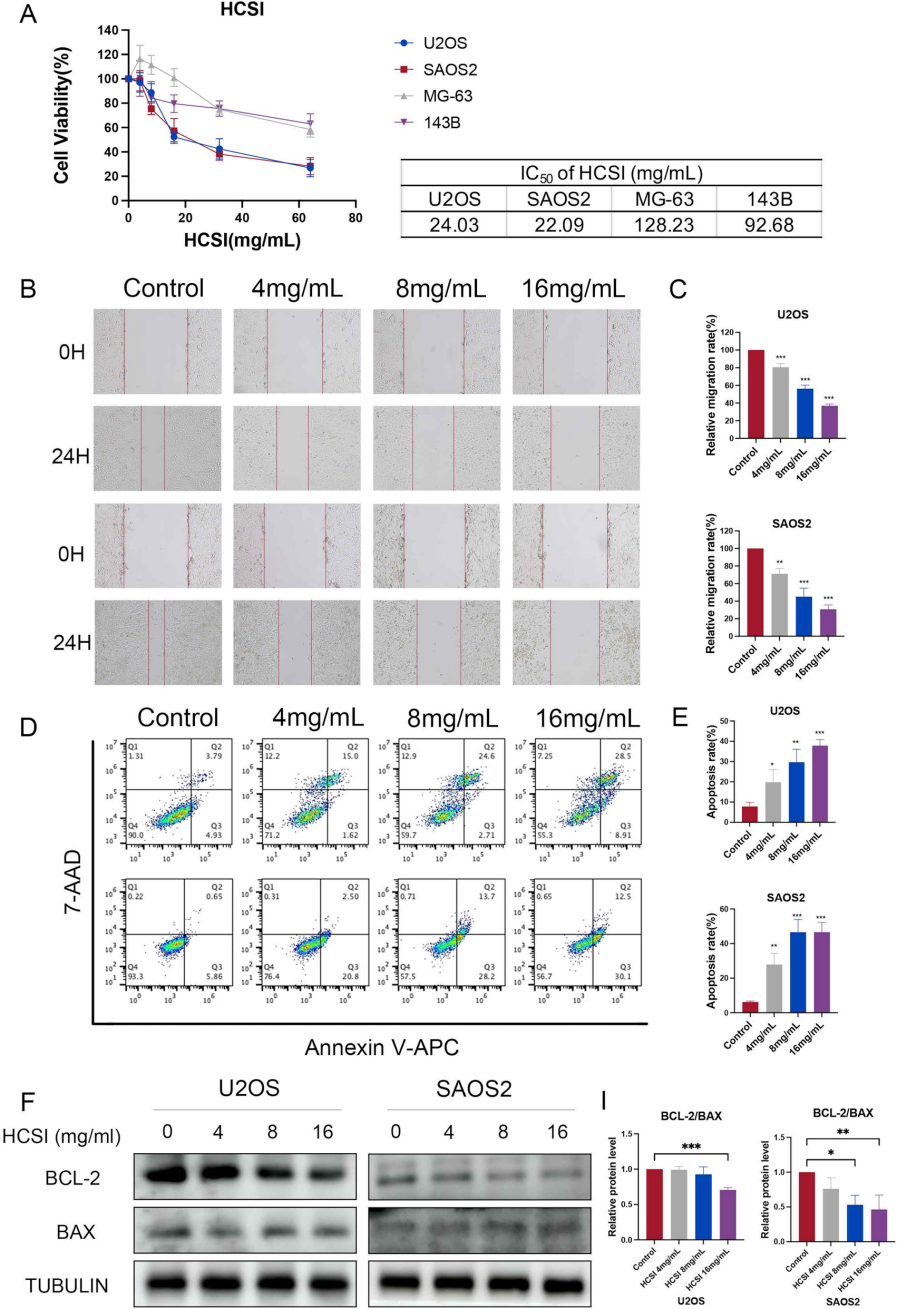
HCSI inhibits the proliferation and migration of osteosarcoma cells in vitro. **A** The CCK-8 assay was used to detect the changes in cell viability after 24 h of HCSI (4, 8, 16, 32 mg/ml) treatment on U2OS, SAOS2, MG-63 and 143B cells, and to screen for the appropriate intervention concentration. **B**-**C** Flow cytometry detected that HCSI (4, 8, 16 mg/ml) induces apoptosis in U2OS and SAOS2 cells. **D**-**E** Wound healing assay was used to determine the migration ability of different concentrations of HCSI on U2OS and SAOS2 cells. **F**-**I** Western blotting was used to detect the expression of proliferative protein Bcl-2 and Bax after 24 h of intervention with different concentrations of HCSI. Data are presented as mean ± SD (n = 3). *P < 0.05, **P < 0.01, and ***P < 0.001 versus the Control group

In order to validate the predicted anti-osteosarcoma effects of HCSI from network pharmacology and bioinformatics analysis, we used CCK-8 assays to evaluate the effect of different concentrations of HCSI (0, 2, 4, 8, 16, and 32 mg/mL) on U2OS, MG-63, SAOS2 and 143B cells for viability assessment at 24 h. As shown in Fig. 8A, HCSI inhibited the proliferation of the above four cells, and we selected SAOS2 and U2OS cells for subsequent experiments. Then, three doses (4, 8, and 16 mg/mL) were selected to interfere with the osteosarcoma cells for 24 h. The results of cell migration experiments showed that HCSI can apparently reduce the wound healing of osteosarcoma cells (Fig. 8B, C). The results of APC Annexin V/7-AAD double staining showed that HCSI significantly induced apoptosis in a dose-dependent manner in U2OS and SAOS2 cells at concentrations of 4, 8, and 16 mg/mL (Fig. 8D, E). In addition, HCSI reduced Bcl-2 protein expression and promoted Bax protein expression, further confirming its role in inhibiting cell proliferation (Fig. 8F).

### HCSI regulated lipid metabolism in osteosarcoma cells

**Fig. 9:**
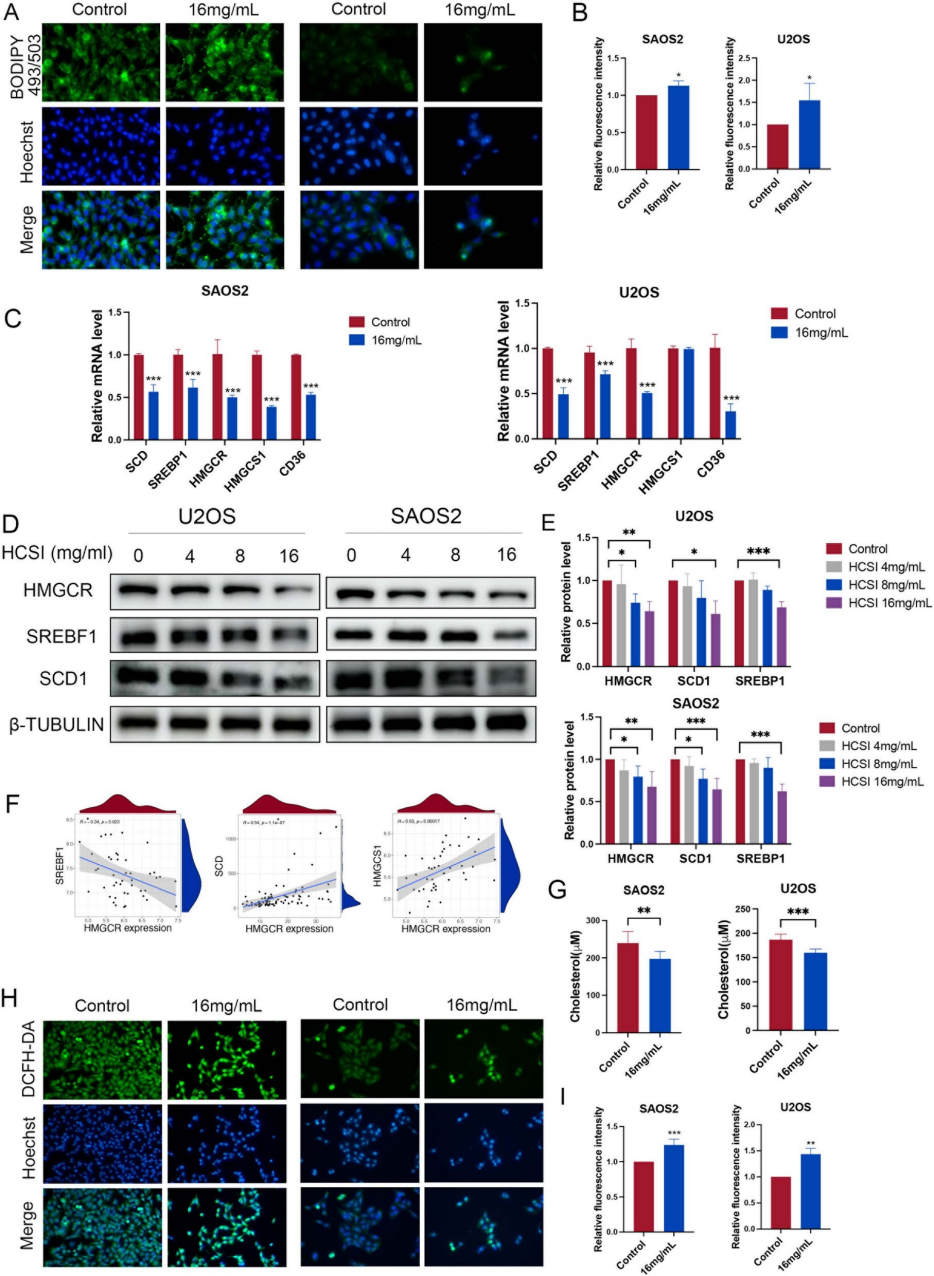
HCSI regulated lipid metabolism in osteosarcoma cells. **A**-**B** Observation of neutral lipid content by staining with fluorescence dye BODIPY 493/503 in U2OS and SAOS2 cells with HCSI treatment. **C** Quantitative RT-PCR for mRNA expression levels of SCD1, SREBF1, HMGCR, HMGCS1 and CD36 with HCSI treatment (n = 4). **D**-**E** Protein levels of SCD1, SREBF1, HMGCR in H U2OS and SAOS2 cells with HCSI treatment. **F** Spearman correlation analysis of the relationship between the mRNA expression levels of HMGCR and SCD1, HMGCS1 and HMGCR in tumor tissues from 42 osteosarcoma patients. **G** Intracellular levels of cholesterol were measured in U2OS and SAOS2 cells with HCSI treatment. **H**-**I** Observation of intracellular ROS levels by staining with fluorescence dye DCFH-DA in U2OS and SAOS2 cells with HCSI treatment. Data are presented as mean ± SD (n = 3). *P < 0.05, **P < 0.01, and ***P < 0.001 versus the Control group

Based on the results of metabolomics and bioinformatics analyses, we found that the inhibition of osteosarcoma cell proliferation by HCSI is related to the regulation of lipid metabolism. Therefore, staining of intracellular neutral lipids with the fluorescent lipophilic dye BODIPY 493/503 was performed in U2OS and SAOS2 cells, which indicated that the content of neutral lipid droplets was positively correlated with HCSI treatment (Fig. 9A-B). Increased lipid content could result from accelerated lipid biosynthesis, fatty acid uptake and catabolism. Thus, the expression levels of key molecules involved in fatty acid synthesis (SCD1, SREBF1), cholesterol biosynthesis (HMGCS1, HMGCR) and fatty acid uptake (CD36) were determined upon HCSI treatment in U2OS and SAOS2 cells. As shown in Fig. 9C, HCSI markedly suppressed the mRNA expression levels of SCD1, SREBF1, HMGCS1, HMGCR and CD36 in SAOS2 cells, while SCD1, SREBF1, HMGCR and CD36 in U2OS cells. In Fig. 9D-E, HCSI suppressed the protein expression of SCD1, SREBF1 and HMGCR in both osteosarcoma cells. Spearman rank correlation analysis revealed significant correlations between the expression levels of HMGCR and SCD1 (r = 0.54, p < 0.01), HMGCS1 (r = 0.53, p < 0.01) and SREBF1 (r = − 0.34, p < 0.05) (Fig. 9F). To further support the function of HMGCR in the aberrant lipid metabolism of osteosarcoma cells, we set out to measure cholesterol levels in SAOS2 and U2OS cells and found that HCSI decreased cholesterol levels in both cells (Fig. 9G). It is well known that lipids maintain membrane biosynthesis and provide an important source of energy under conditions of metabolic stress [36]. We then investigated the levels of reactive oxygen species (ROS) in U2OS and SAOS2 cells. The results showed that HCSI increased ROS production (Fig. 9H-I). Taken together, these data strongly suggest that HCSI plays a dominant role in the regulation of lipid metabolism in OST. Collectively, these data indicate that HCSI plays a prominent role in regulating lipid metabolism in osteosarcoma cells and profiles energy metabolism along with redox homeostasis.

### HCSI exerts inhibition of osteosarcoma cell progression by regulating HMGCR

**Fig. 10:**
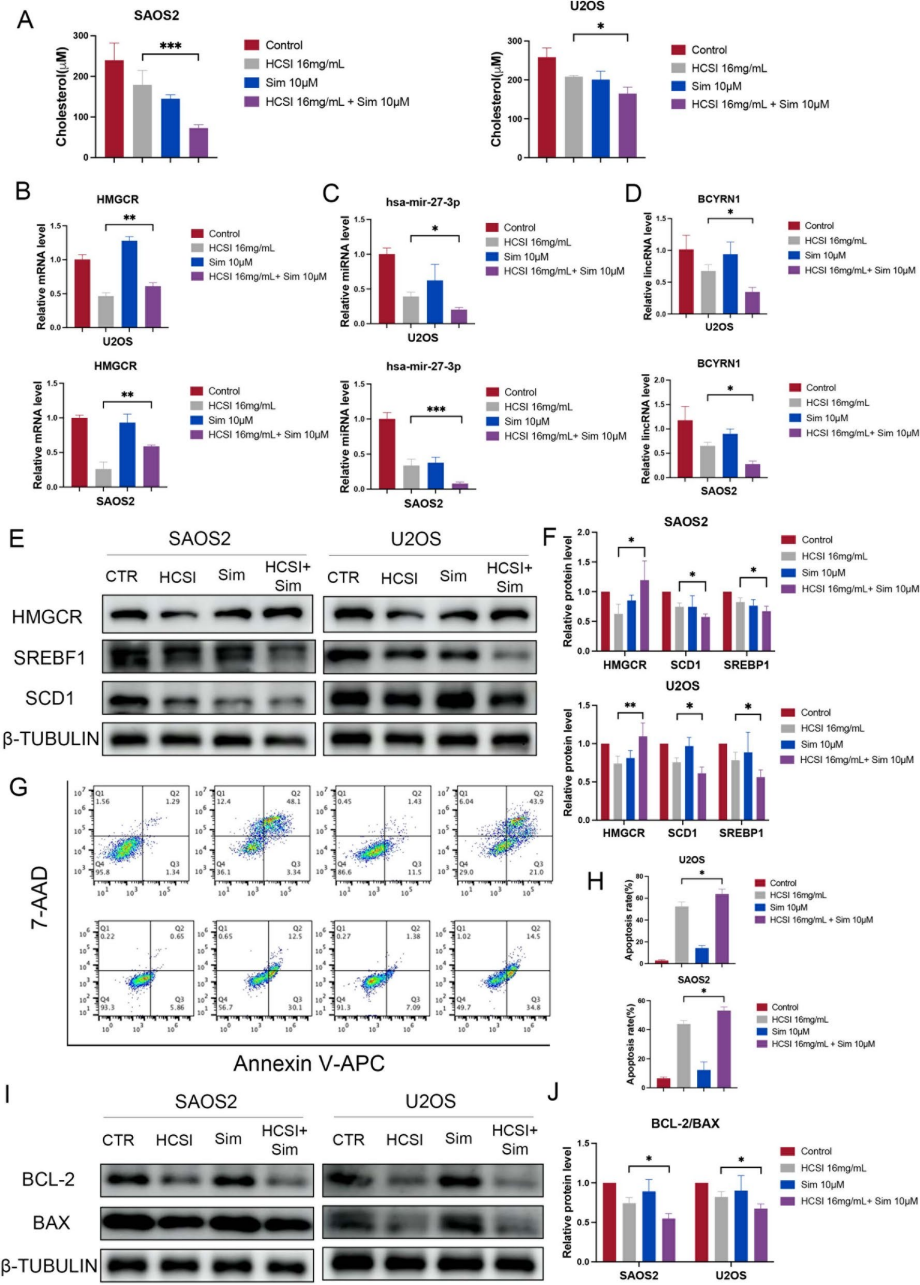
HCSI regulated lipid metabolism in osteosarcoma cells. **A** Intracellular levels of cholesterol were measured in U2OS and SAOS2 cells with HCSI and simvastatin treatment. **B**-**D** Quantitative RT-PCR for mRNA, miRNA and lncRNA expression levels of BCYRN1, miR-27a-3p and HMGCR with HCSI and simvastatin treatment. (n = 4). **E**–**F** Protein levels of SCD1, SREBF1, HMGCR in the U2OS and SAOS2 cells with HCSI and simvastatin treatment. **G**-**H** Flow cytometry detected that HCSI and simvastatin induces apoptosis in U2OS and SAOS2 cells. **I**-**J** Western blotting was used to detect the expression of proliferative protein Bcl-2 and Bax after 24 h of intervention with HCSI and simvastatin treatment. Data are presented as mean ± SD (n = 3). *P < 0.05, **P < 0.01, and ***P < 0.001 versus the Control group

To further investigate the effect of HMGCR on osteosarcoma cell proliferation, we used simvastatin, which is a hypocholesterolemic drug that blocks the activity of the HMGCR receptor [37]. As shown in Fig. 10A, the cholesterol levels of SAOS2 and U2OS cells were further reduced by the addition of simvastatin. We then examined the expression of the mRNA (Fig. 10B), miRNA (Fig. 10C), lncRNA (Fig. 10D) and protein with the addition of simvastatin. We found that the inhibition of miR-27a-3p and BCYRN1 was more significant in the simvastatin group, while the inhibition of HMGCR was reversed. Western blot results revealed that the simvastatin group further reduced the protein levels of SCD1 and SREBF1, whereas the opposite effect was exhibited on HMGCR (Fig. 10E, F). These results suggest that HCSI may regulate intracellular lipid metabolism through the modulation of HMGCR, which may be induced by the BCYRN1-miR-27a-3p-HMGCR axis. Furthermore, simvastatin significantly increased the apoptosis rate of SAOS2 and U2OS cells compared to the HCSI group (Fig. 10G, H). Western blot analysis further demonstrated that simvastatin group promoted the expression of Bax and inhibited the expression of Bcl-2 compared to the HCSI group (Fig. 10I-J). Taken together, these results suggest that HCSI exerts an anticancer effect in the osteosarcoma cells through the modulation of HMGCR.

## Discussion

Osteosarcoma is a highly aggressive tumor that lacks effective therapeutic targets. Despite extensive research in recent decades, new clinical regimens capable of improving osteosarcoma patient prognosis remain elusive. TCM is one of the research hotspots in cancer therapy due to its novel pharmacological mechanism, low toxicity and limited side effects [38]. TCM injection HCSI exerts remarkable therapeutic effects on different tumors. It can delay hepatocellular carcinoma progression by regulating lipid metabolism via SREBP1 signaling pathway [16]. Additionally, the active ingredients of HCSI inhibit tumor progression by enhancing FOXO1-mediated transcription of FCGBP in osteosarcoma. In this study, metabolomics, network pharmacology and bioinformatics were applied to reveal the potential molecular mechanisms by which HCSI regulates lipid metabolism in osteosarcoma [39].

Metabolic reprogramming has been identified as a hallmark of cancer, and the important role of dysregulated lipid metabolism in cancer is increasingly recognized [40]. According to the results of non-target metabolomics analysis, a total of 209 differential metabolites after HCSI treatment in SAOS2 cells were identified, with enriched pathways closely related to lipid metabolism. Tumor tissues undergo lipid reprogramming through aberrant increases in multiple pathways, including de novo lipid synthesis, exogenous uptake, cholesterol synthesis and lipid oxidation. Primary lipid metabolites involved in the analysis in this study included sphinganine, palmitic acid and L-palmitoylcarnitine. The sphingosine can be converted into an intermediate of palmitic acid [41]. It has been reported that palmitic acid treatments can increase SREBP2-mediated cholesterol accumulation in HepG2 cells [42]. Therefore, the anti-osteosarcoma mechanism of HCSI may be related to the regulation of lipid metabolism and modulation of cholesterol levels.

Based on the findings from metabolomics analysis, network pharmacology was employed to predict the active ingredients and targets of HCSI. Cinobufotalin, cinobufagin, telocinobufagin, gamabufotalin, arenobufagin, bufotalin, resibufagenin and bufalin were identified as the primary bioactive compounds in HCSI. It has been previously demonstrated that cinobufotalin can suppress proliferation of hepatocellular carcinoma by interfering with de novo lipid synthesis [43]. Meanwhile, arenobufagin can modulate cholesterol metabolism to inhibit hepatocellular carcinoma progression [44]. Bufalin can mediate adipogenesis via SREBF1 [45]. 44 key targets including HMGCR, SIRT2, HIF-1A, BACE1 were screened. These results indicate that HCSI may regulate lipid metabolism through the synergistic actions of multiple active ingredients.

However, lipid metabolism is more than a self-regulation network. Advancements in human genome technology have uncovered noncoding RNAs as a novel class of regulators in lipid metabolism [46]. LncRNAs and miRNAs are proved to be critical regulators during the lipid metabolism process and lipid signaling [47, 48]. By exploring the differential expression of lncRNAs and miRNAs in paracancerous and tumor tissues of osteosarcoma patients, the transcriptional regulation of lncRNA-miRNA-mRNA was summarized in this study. The ceRNA network was constructed by integrative analysis with targets obtained from network pharmacology and bioinformatics, including 8 mRNAs, 22 miRNAs and 69 lncRNAs. This indicates that HCSI may influence the lipid metabolism through the complicated connection between ceRNA.

Combining machine learning and in vitro experiments, HMGCR was finally focused with miR-27a-3p and BCYRN1. The results of molecular docking also demonstrated that the active monomers of HCSI bind stably to HMGCR. BCYRN1 can regulate target genes to promote tumor progression by competing with various miRNAs. It has been proved that the lncRNA BCYRN1 affected the occurrence and development of colorectal cancer by regulating the effects of miR-204-3p on KRAS. BCYRN1 can also inhibit glioma progression by sponging miR-619-5p [49]. miR-27a-3p, a tumor promoter, is significantly overexpressed in osteosarcoma. It has been found that down-regulation of miR-27a-3p can inhibit the proliferation and invasion of osteosarcoma cells [50]. Furthermore, it is reported that miR-27a-3p achieves regulation of lipid metabolism through binding the 3'UTR of HMGCR [31]. In this study, we firstly found that BCYRN1 may interact with miR-27a-3p through a competitive endogenous RNA mechanism. However, the specific mechanism still needs to be further investigated.

To further confirm the role of HCSI in inhibiting osteosarcoma progression by lipid regulation, we performed in vitro pharmacological experiments. HCSI obviously inhibited cholesterol level by downregulating the mRNA and protein level of HMGCR. Simultaneously, HCSI also reduced the expression of SCD1 and SREBP1. The results indicate HCSI inhibited the physiological processes of de novo fatty acid synthesis and cholesterol biosynthesis in osteosarcoma cells. However, in our current study, we did not observe a relevant effect of HCSI on fatty acid oxidation in osteosarcoma cells. This suggests that HCSI may play a key role in osteosarcoma progression through lipogenesis rather than lipolysis. HMGCR, together with HMGCS1, is the rate-limiting enzyme in the mevalonate pathway, which is the main pathway for cholesterol biosynthesis [51]. Targeting HMGCR with statins is considered to be a safe and clinically relevant anti-tumor approach [52, 53]. Statins have been shown to stimulate their anticancer effects by depleting cholesterol [54]. A study has shown that simvastatin decreases cholesterol levels in lipid rafts in prostate cancer cells, thereby impeding AKT signaling and inducing apoptosis [55]. Consistently with these results, our study found that HCSI in combination with simvastatin reduced intracellular cholesterol levels more effectively than HCSI alone. Therefore, the combination enhanced the level of apoptosis in osteosarcoma cells and down-regulating the expression of Bcl-2/Bax by HMGCR-miR-27a-3p-BCYRN1 axis. However, statin application triggers negative feedback and activates the mevalonate pathway, which leads to the upregulation of HMGCR [56]. This explains why the combination of HCSI with simvastatin reversed the expression of HMGCR instead.

RNA sequencing (RNA-seq) has been used as an efficacious instrument for the investigation of osteosarcoma because it provides an overview of biological components and facilitate the identification of novel genes [57]. In the subsequent research, RNA-seq will be employed with metabolomics to enable multi-omics studies of mechanisms and targets in osteosarcoma. In conclusion, our results confirmed that the HCSI may be a potential strategy for inhibiting osteosarcoma progression.

## Conclusion

In summary, this study indicated that HCSI could regulate lipid metabolism by modulating HMGCR ceRNA axis and cholesterol level to exerts anti-osteosarcoma effect by inducing osteosarcoma cell apoptosis. The integration of metabolomics, network pharmacology and bioinformatics in this study have provided a plausible mechanism for HCSI inhibiting osteosarcoma progression and highlighted HCSI as a potential promising medicine for osteosarcoma. Additionally, the regulation of lipid metabolism related to ceRNA network might also constitute a novel strategy for the treatment on osteosarcoma.

## Supplementary Information

Additional file 1.

Additional file 2.

Additional file 3.

Additional file 4.

Additional file 5.

## Data Availability

All the data generated and analyzed during this study are available from the corresponding author.

## Abbreviations

LncRNA: Long noncoding RNA
MiRNA: MicroRNA
mRNA: Messenger RNA
qRT-PCR: Quantitative real-time polymerase chain reaction
ANOVA: One-way Analysis of Variance
OPLS-DA: Orthogonal partial least squares discriminant analysis
DEGs: Differentially expressed genes
SREBF1: Sterol regulatory element binding transcription factor 1
FASN: Fatty acid synthase
ceRNA: Competing endogenous RNAs
GO: Gene Ontology
KEGG: Kyoto Encyclopedia of Genes and Genomes
Lasso: Least absolute shrinkage and selection operator
RF: Random Forest
SVM-RFE: Support Vector Machine Recursive Feature Elimination
HMGCR: 3-Hydroxy-3-methylglutaryl-CoA reductase
BCYRN1: Brain Cytoplasmic RNA 1
SCD1: Stearoyl-CoA desaturase 1
BCL-2: B-cell lymphoma 2
BAX: Bcl-2 associated X
